# Psychosocial Factors That Influence Occupational Stress in Operating Room Nurses’ Working Environment: A Scoping Review

**DOI:** 10.1177/21650799251377451

**Published:** 2025-11-06

**Authors:** Vibeke Tjugum, Lena Rengård Kolstad, Marie Hamilton Larsen, Simen A. Steindal

**Affiliations:** 1 Lovisenberg Diaconal University College; 2 Institute of Nursing, VID Specialized University

**Keywords:** operating room nurse, psychosocial factors, occupational stress, review, work environment

## Abstract

**Background:**

Operating room nurses play complex roles and are faced with substantial demands, including ensuring patient safety, managing interprofessional collaboration, and adapting to unpredictable challenges. These factors contribute to occupational stress influenced by psychosocial factors, such as workload, interpersonal conflicts, and time pressures. While prior reviews have explored stress in the operating room, none have focused specifically on the psychosocial factors that impact operating room nurses. This scoping review aimed to map existing research on these factors and to identify knowledge gaps and inform future studies.

**Methods:**

This scoping review followed the methodological framework given by Arksey and O’Malley. CINAHL, EMBASE, MEDLINE, PsycINFO, and Web of Science were searched from inception until October 11, 2024. Peer-reviewed studies in English or Scandinavian languages were included if they reported psychosocial factors influencing occupational stress in operating room nurses working environment and employed qualitative, quantitative, multimethod, or mixed-method designs.

**Findings:**

From the 36 papers included, data were organized into three thematic groups: (a) interprofessional factors contributing to occupational stress, (b) work-related factors influencing occupational stress, and (c) stress levels and personal factors influencing occupational stress among operating room nurses.

**Application to Practice/Conclusions:**

The primary psychosocial factors contributing to occupational stress among operating room nurses included interprofessional challenges and high workload. Work-related moral distress associated with patient care complexities and safety also played a significant role. These findings highlight a need for strong leadership, improved team dynamics, and supportive interventions to manage stress.

## Background

The role of operating room nurses’ (ORNs) involves managing the complexities within the operating room (OR) to ensure a seamless surgical process (Göras et al., 2020). The execution and safety of surgical procedures is heavily dependent on seamless collaboration and communication among healthcare professionals in OR teams (Chellam Singh & Arulappan, 2023). Hence, ORNs are pivotal in the planning and preparation phases of surgery, coordinating the flow of information in the OR and adapting to unforeseen challenges (Göras et al., 2020). ORNs often face challenges and work demands, such as handling patients’ conditions, time constraints, emergency situations, and collaboration with multidisciplinary healthcare professionals (Rodrigues et al., 2020). ORNs’ workdays are unpredictable and challenging, and their tasks often demand that they are flexible and adapt quickly to a work environment perceived as stressful (McGarvey et al., 2000).

Given these conditions, ORNs are exposed to psychosocial factors that lead to occupational stress (Nieuwenhuijsen et al., 2010), including cognitive, emotional, behavioral, and relational aspects that are pivotal in influencing employees’ health and performance (Marmot et al., 1999). Psychosocial factors are occupational stress factors that affect general health outcomes (Niedhammer et al., 2008) and can lead to burnout, substance abuse, and even suicide among health care professionals (Maslach & Leiter, 2016). Multiple theories have been developed to explore this area and provide frameworks to understand the complex nature and influence of occupational stress. Prominent among these is the job demand–control support model (Karasek & Theorell, 1990), which assumes that health may be negatively affected by job demands and positively affected by control and social support in the workplace. The person–environment fit model (French et al., 1982) states that stress occurs when there is a mismatch between an individual’s background, capability, and job demands, while the transactional model of stress and coping (Lazarus & Folkman, 1984) focuses on how individuals appraise and cope with stressors.

Previous reviews examined diverse aspects of the OR psychosocial work environment. Three reviews examined various viewpoints on stress (Brandão & Galvão, 2013; Chrouser et al., 2018; Jacob, 2015). Brandão and Galvão (2013) found that workplace stress negatively impacted perioperative nursing teams’ health and affected their professional and personal lives. Chrouser et al. (2018) explored how intraoperative stressors affect surgical performance and developed a conceptual framework for surgical stress. Jacob (2015) identified interprofessional conflicts, lack of resources, time constraints, workload, and the nature of the work as the main sources of ORNs’ occupational stress.

Other reviews examined job satisfaction, emotions, and team communication in the OR (James-Scotter et al., 2019; H. Lee et al., 2023). James-Scotter et al. (2019) identified multiple factors influencing OR team job satisfaction, including personal characteristics, setting and specialty, clinical roles, job control, team dynamics, work conditions, career development, research opportunities, and organizational management. H. Lee et al. (2023) found that hierarchical culture and OR leadership expectations contributed to negative emotional experiences, adversely affecting team functions and communication.

While previous reviews examined certain aspects of the psychosocial work environment in the OR, they either included a broad spectrum of OR personnel or focused on general or specific stress factors within the OR without mapping the specific psychosocial factors faced by ORNs. Thus, there is a notable lack of comprehensive understanding of how psychosocial factors influence ORNs’ work environment. Focusing on these factors and solely on ORNs could enhance our understanding of the sources of their occupational stress. A scoping review could also identify research gaps and establish future research priorities. To our knowledge, no scoping review has systematically mapped existing research addressing the psychosocial factors that influence occupational stress within ORNs’ working environment.

This review aimed to systematically map published studies on the psychosocial factors influencing occupational stress in ORNs’ working environments and to identify knowledge gaps and inform future studies.

## Methods

This scoping review employed Arksey and O’Malley (2005) methodological framework. The review was reported in accordance with the Preferred Reporting Items for Systematic Reviews and Meta-Analyses Extension for Scoping Reviews checklist (Tricco et al., 2018).

### Search Methods

A systematic search was conducted on the CINAHL Complete (EBSCOhost), EMBASE (Ovid), MEDLINE All (Ovid), PsycINFO (Ovid), and Web of Science databases for peer-reviewed studies published from inception until October 11, 2024. The database searches were limited to papers published in English, Swedish, Danish, and Norwegian. The Medical Subject Headings (MeSH) terms used in MEDLINE were a combination of the following: (operating room nurse OR perioperative nurse OR operating rooms) AND (exp stress, psychological OR stress, physiological). Search terms related to Population and Context were combined with OR to minimize the risk of missing relevant studies, and search terms describing the Concept were combined with OR. These search sets were then further combined with relevant textwords using Boolean operators (AND, OR). The search strategy used for MEDLINE (Table 1) was adapted to other databases. A second research librarian reviewed the search strategy for all the databases using the Peer Review of Electronic Search Strategies checklist (McGowan et al., 2016). We also searched relevant journals and reference lists of included manuscripts for additional relevant peer-reviewed studies.

**Table 1.** Search Strategy in MEDLINE All for Examining Operating Room Nurses Psychosocial Factors and Occupational Stress

1	Operating Room Nursing/
2	Perioperative Nursing/
3	Operating Rooms/
4	(RN adj2 “first assistant*”).tw,kf.
5	((nurse or nurses or nursing* or staff* or personnel* or team or teams or clinician* or professional or professionals or practitioner* or employee*) adj3 (operating or operative or operation or operatory or perop* or per-op* or periop* or peri-op* or interop* or inter-op* or intraop* or intra-op* or theater* or theatre* or surgical or surgery or scrub* or circulating or scout or “first assistant*”)).tw,kf.
6	(((operating or operative or operation or operatory or surgical or surgery) adj4 (room* or theater* or theatre* or unit or units or suite* or department* or ward* or clinic or clinics or center* or centre* or division*)) and (nurse or nurses or nursing* or staff* or personnel* or team or teams or clinician* or professional or professionals or practitioner* or employee*)).tw,kf.
7	1 or 2 or 3 or 4 or 5 or 6
8	exp Stress, Psychological/
9	Stress, Physiological/
10	(stress or stresses or stressed or stressor* or stressful).tw,kf.
11	(burnout or burn-out).tw,kf.
12	8 or 9 or 10 or 11
13	7 and 12
14	limit 13 to (danish or english or norwegian or swedish)

Two pairs of authors independently assessed title, abstract, and full text, whether each publication met the eligibility criteria. In cases of disagreement regarding inclusion, a third author conducted an independent assessment; the final decision was based on consensus.

### Inclusion and Exclusion Criteria

The inclusion and exclusion criteria were developed using the Population, Concept, and Context (PCC) framework. The population of interest was ORNs. The concept focused on psychosocial factors that influence occupational stress, including related concepts such as work-related stress, job stress, and stress-related outcomes. The context was limited to the operating room work environment. Eligible studies included empirical peer-reviewed research using qualitative, quantitative, multimethod, or mixed-methods designs and published in peer-reviewed journals.

Papers were excluded if they focused on healthcare professionals other than ORNs (e.g., students, trainees, patients, or nursing leaders), addressed only physical factors, were conducted outside of the operating room setting, or were not empirical, peer-reviewed research (e.g., theses, editorials, letters, comments, conference abstracts or proceedings, books, reports, review articles, research protocols, or guidelines). These criteria were piloted by the first and second authors on 100 publications.

### Data Abstraction

A standardized data collection form developed in Microsoft Excel was used for data extraction from the included papers. The form included author, year of publication, and country; study design; study aim; sample size; setting; outcome measure; psychosocial factors; and findings. One author extracted the data, while the other author validated its accuracy against the papers, making approvals or modifications as necessary.

### Synthesis

To address the aim, an inductive approach was employed to organize and thematize the findings from the included papers (Steindal et al., 2020). The results section of each paper was read several times to identify frequent patterns across the papers in terms of psychosocial factors influencing occupational stress in ORNs’ working environment. NVivo software was used to organize the findings and develop a thematic structure (Allsop et al., 2022). The data were systematically organized into three thematic groups. Subsequently, the authors engaged in a comprehensive discussion of the findings. A consensus was reached among all authors regarding the definitive allocation of findings into their respective thematic groups.

## Results

**Figure 1.**
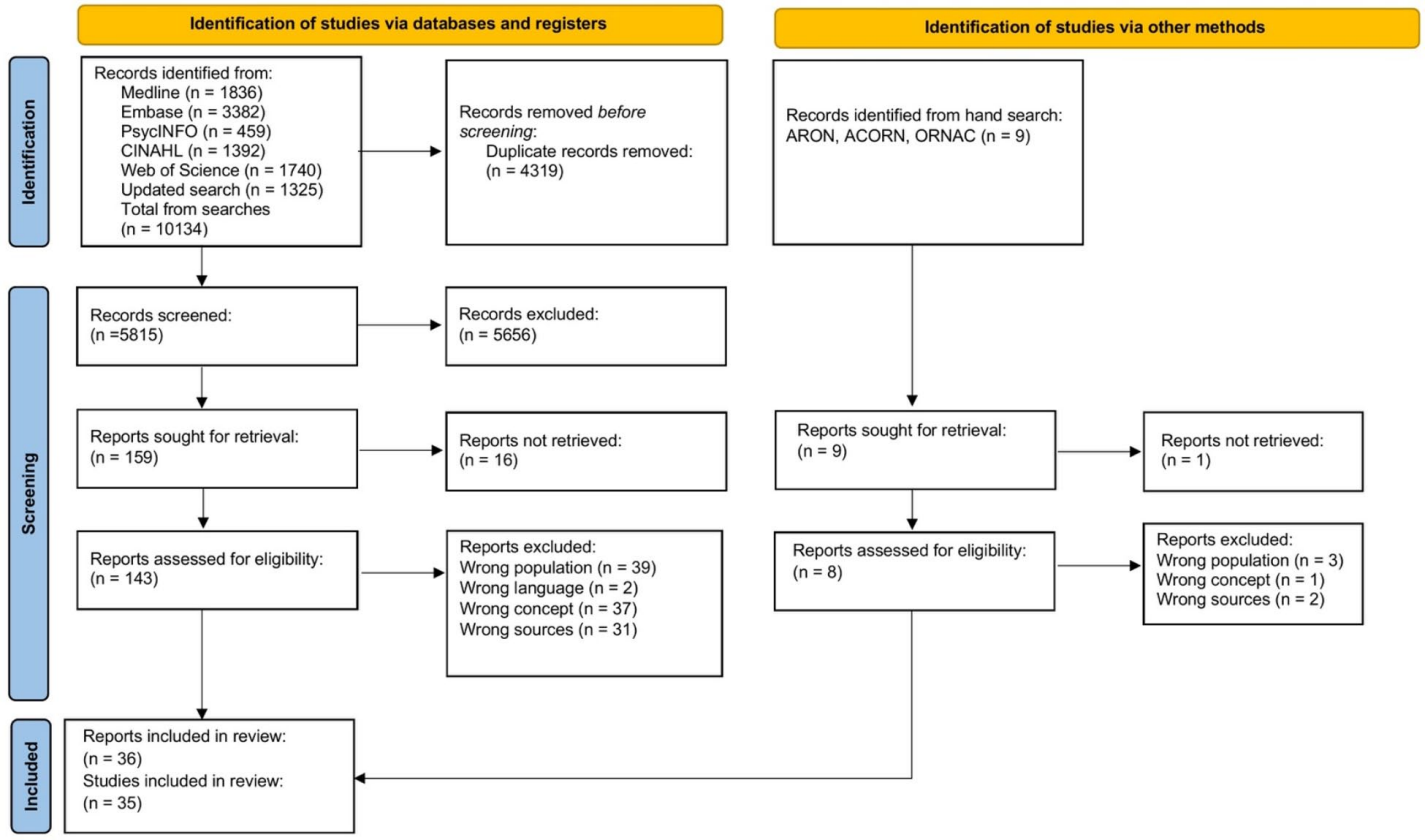
The PRISMA 2020 flow diagram for a summary of the selection of sources

The database searches yielded 10,134 publications. After 4,319 duplicates were eliminated, the titles and abstracts of 5,815 publications were screened. Based on the inclusion and exclusion criteria, the full text of 143 publications was read, and 34 papers from 33 studies were included. A manual search identified two additional studies. In total, 36 papers and 35 studies were included in the review (Figure 1).

The characteristics of the included papers are presented in Table 2. The papers originated from 19 different countries across six continents and were published between 1977 and 2023. A qualitative design was utilized in nine of the studies, while a quantitative approach was employed in 27 studies.

**Table 2.** Characteristics of the Included Papers on Psychosocial Factors That Influence Occupational Stress in Operating Room Nurses’ Working Environment

Author (publication year), country	Study design	Study aim	Sample size setting	Psychosocial factors	Findings
Ackah and Kwashie (2023), Ghana	Individual semi-structured interviews, exploratory descriptive	To explore events from outside and within the OR of a teaching hospital in Ghana that caused stress for the nurses.	12 ORNs; gender: NR; age: *M* 34 (*SD* 7.2, range 27–51); education level: 58% diploma in nursing; shift schedule: NR; work experience: NR Teaching hospital; surgical areas: general, genitourinary, maxillofacial, eye	Interprofessional, work-related, personal	The sources of stress were identified as either internal or external. Internal sources were linked to individual behavior patterns. External sources were related to daily hassles, job demands, workplace resources, coworkers, and moral distress.
Aholaakko (2011), Finland	Qualitative stimulated recall interviews	To explore aseptic practice-related stress in surgery. The objective was to define stress-related factors and the means to reduce stress.	31 ORNs; gender: 28 female; age: 20–60 years; education level: all ﬁrst-level registered nurse qualiﬁcation; shift schedule: NR; work experience: 2 months >30 years University central hospital; surgical area: breast	Interprofessional, work-related, personal	Aseptic practice-related stress was constructed in 16-level categories. The study identified connections between qualitatively attributed personnel and seven stress factors—working experience, time, equipment, person, patient, working morals, and power.
Akansel et al. (2019), Turkey	Cross-sectional, descriptive	To determine the association between organizational stress and fatigue in ORNs.	46 ORNs; gender: 40 female; age: *M* 35.4 years (*SD* 6.3); education level: 73.9% graduate (MSc); shift schedule: 69% rotating shifts; work experience: *M* 11.4 years (*SD* 7.5) University hospital; surgical area: NR	Work-related, personal	Social support and skill use were among the highest scores for organizational stress. There was an association between fatigue and organizational stress levels and factors such as work demands, skill use, and social support. ORNs with chronic diseases reported higher levels of stress. Contributing factors for fatigue were demanding work, stress, understaffing, and working overtime.
Akgül and Aksoy (2021), Turkey	Descriptive correlation	To determine the relationship between organizational stress levels and patient safety attitudes among OR staff.	46 ORNs; gender: 33 female; age: ≥20 years; education level: NR; shift schedule: NR; work experience: ≥3 months University hospital; surgical areas: pediatric, general, breast, eye, gynecological and obstetric, cardiovascular, otorhinolaryngologic, brain, orthopedics and traumatology, plastic and reconstructive, urologic	Personal	Surgeons reported higher sources of stress related to the nature of their work and management than ORNs. ORNs reported more financial stress than anesthetists and OR staff. OR staff had higher stress recognition and overall SAQ-OR scores than ORNs.
Almodibeg and Smith (2021), Saudi Arabia	Cross-sectional, descriptive	To identify the level of burnout and its most significant causes among perioperative nurses.	39 ORNs; gender: 89.7% female; age: NR; education level: NR; work experience: 35.8% junior nurse Regional hospital; surgical area: NR	Interprofessional, work-related, personal	High levels of emotional exhaustion (87.2%) and depersonalization (56.4%) were detected among the ORNs. The ORNs showed a low sense of personal accomplishment (15.4%) and 5% had burnout. High workload, lack of departmental support, undesirable supervision, staff shortage, poor teamwork, insufficient salary, and occupational hazards were reasons for burnout.
Bianchi (2008), Brazil	Cross-sectional, descriptive	To describe stressors and evaluate stress levels among ORNs.	80 ORNs; gender: 93% female; age: 95% >50 years; education level: 21.25% specialized ORNs; shift schedule: NR; work experience: 67.5% <15 years High-complexity hospitals; surgical areas: cardiovascular, neurological, trauma, transplant	Work-related, personal	Stress levels of ORNs were moderate. Functioning of the unit, unit management, and working conditions were the most stressful areas. Almost half of the ORNs reported high stress levels caused by “performing duties with minimal time” as part of working conditions. Additionally, 33.75% of ORNs found “facing the death of a patient” in the area of nursing care to be highly stressful.
Chen et al. (2009), Taiwan	Cross-sectional	To determine the stressors, stress coping strategies, and job satisfaction of nursing staff working in the OR and to evaluate the influence of demographic characteristics on job stress, stress coping strategies, and job satisfaction.	112 ORNs; gender: 112 female; age: *M* 32 years (*SD* 5.8); education level: 57% junior college; shift schedule: 92% shift; work experience: 1–>20 years Regional teaching hospitals, community teaching hospitals; surgical area: NR	Work-related, personal	A link was identified between the type of hospital in which the ORNs worked and their stress levels and job satisfaction. Patient safety stood out as the primary stressor among ORNs, with administrative feedback being the most frequently reported stressor.
Cook et al. (2001), USA	Nonexperimental, descriptive	To explore the incidence and impact of verbal abuse by physicians on perioperative nurses.	78 ORNs; gender: 76 females; age: *M* 44.0 years (*SD* 8.1); education level: 61% certified ORNs; shift schedule: NR; work experience: *M* 16.0 years (*SD* 8.5) Teaching hospital, community hospital, ambulatory care center; surgical area: NR	Interprofessional	The five types of verbal abuse that caused the most stress were identified as abusive anger, condescension, accusing and blaming, judging and criticizing, and blocking and diverting. The three most negative effects of verbal abuse were a strained relationship with the physician, reduced job satisfaction, and diminished well-being in the workplace.
Cronin-Stubbs and Rooks (1985), USA	Correlation, descriptive survey	To identify stressors associated with burnout among critical care, psychiatric, OR, and medical nurses.	65 ORNs; gender: NR; age: NR; education level: NR; shift schedule: NR; work experience: NR Medical center hospital; surgical area: NR	Personal	Critical care nurses and medical nurses encountered occupational stressors more frequently and intensely than ORNs. ORNs experienced significantly more help than psychiatric nurses. There was no difference in burnout among the groups.
Elfering et al. (2017), Germany/Switzerland	Cross-sectional	To test whether cognitive stress symptoms are positively associated with emotional abuse, emotional- and task-related demands, and resources in surgery work.	48 ORNs; gender: 81.3% female; age: *M* 36.4 years (*SD* 10.6); education level: 45.8% apprenticeship; shift schedule: NR; work experience: NR Type of hospital; NR; surgical area: NR	Interprofessional, personal	Emotional demands and abuse were confirmed as risk factors for cognitive stress symptoms, including concentration issues, decision-making difficulties, and memory impairment. Cognitive stress symptoms were positively associated with emotional demands and emotional abuse.
Eskola et al. (2016), Finland	Cross-sectional	To investigate the workplace culture in the OR and assess the factors associated with it.	98 ORNs; gender: 92 female; age: *M* 38 years (range 23–62); education level: 93% registered nurses; shift schedule: 88% day; work experience: 60% >10 years University hospitals, local hospitals; surgical areas: general, neuro, day surgery	Work-related, personal	ORNs viewed their workplace culture positively, with low job stress. Job stress was mainly due to workload. Factors such as unit type, years in the current OR, and primary role affected job stress perceptions. Older nurses (45–62 years) reported more stress related to preparation and conflicts compared to younger nurses. Shift work reduced job stress linked to workload and work–social balance.
Gillespie et al. (2013), Australia	Ethnography	To describe team communication and the ways it fostered or threatened safety culture in surgery.	13 ORNs; gender: NR; age: NR; education level: NR; shift schedule: NR; work experience: NR Tertiary care facility; surgical areas: ENT vascular, cardiac, general, ophthalmology, neuro, faciomaxillary, plastics, urology, orthopedic	Interprofessional	Managing contextual stressors in a hierarchical environment was highlighted as important. Providing backup for colleagues was considered a valued aspect of teamwork to ensure that operative procedures ran smoothly and without incident.
Higgins and Macintosh (2010), Canada	Qualitative, descriptive study	To explore ORNs’ perceptions of the effects of physician-perpetrated abuse on their health and ability to provide patient care.	10 ORNs; gender: female; age: 28–58 years (*SD* 50); education level: registered nurse; shift schedule: NR; work experience: >10 years Community hospitals, regional hospitals; surgical area: NR	Interprofessional, personal	Abuse in the OR was linked to three categories: the culture of the OR, including the environment and hierarchy; catalysts such as nurses’ roles, experience, resources, and relationships with physicians; and perceived effects, including psychological, physical, and social health impacts on ORNs.
Holmes et al. (2020), Norway	Qualitative, descriptive study	To explore Norwegian ORNs’ perceptions of how team skills in the interprofessional OR team influence perioperative nursing in relation to patient safety.	10 ORNs; gender: 9 female; age: 40–51 years; education level: registered nurses with postgraduate specialist training; shift schedule: NR; work experience: 5–11 years General hospital, university hospital; surgical area: NR	Interprofessional	ORNs’ work environment, encompassing confidence, stress, energy use, job satisfaction, and poor team skills, such as misunderstandings, interruptions, and a lack of trust increased stress. Good team skills were seen to reduce stress.
Hull et al. (2011), UK	Prospective cross-sectional	To assess teamwork and stress levels experienced by OR team members.	*n* = NR; gender: NR; age: SN *M* 32.4 years (*SD* 6.1), CN *M* 42.4 years (*SD* 9.6); education level: NR; shift schedule: NR; work experience: SN *M* 5.5 years (*SD* 8.2), CN *M* 8.9 years (*SD* 6.8) University teaching hospital; surgical area: gastrointestinal	Work-related	CN were most likely to experience high stress preoperatively but were the least likely to experience this intraoperatively. SN were the least likely to report high stress levels both preoperatively and postoperatively.
Johnstone (2000), Australia	Exploratory survey	To determine the intensity of ORNs’ perceptions of the effects of changes in OR technologies on the nature and volume of their work, job satisfaction and work-related stress.	353 ORNs; gender: NR; age: NR; education level: NR; shift schedule: NR; work experience: NR Type of hospital: NR; surgical area: NR	Work-related	A source of stress among instrument and circulating nurses was role conflict resulting from the incompatible goals of maintaining professional standards and compliance with managerial pressure to “push the cases through.” Moreover, increased technology-related stress was reported among 292 ORNs.
Kawano (2008), Japan	Cross-sectional	To examine the degrees of job-related stress factors and mental and physical symptoms among Japanese hospital nurses in various departments and to clarify the associations between departments and job-related stress factors.	92 ORNs; gender: NR; age: NR; education level: NR; shift schedule: NR; work experience: NR Type of hospital: NR; surgical area: NR	Work-related, personal	ORNs had a significantly lower score in terms of job control and job fitness than other departments. ORNs had lower vigor scores. Working in the OR was linked to lower vigor and higher fatigue and depression, after adjusting for age, marital status, and other factors.
Laflamme et al. (2019), Canada	Ethnography	To describe interprofessional relations to understand their impact on nurse retention while considering the context of OR culture.	11 ORNs; gender: NR; age: NR; education level: NR; shift schedule: NR; work experience: ≥1 year University hospital; surgical area: NR	Interprofessional, personal	Poor team skills—such as misunderstandings, interruptions, and not being able to trust others to do their job properly—created stress. Recognition was linked to feeling respected and appreciated. A certain type of personality made it easier to adapt to the OR.
E. Y. Lee et al. (2022), South Korea	Cross-sectional	To determine the relationships between job stress, resilience, and communication competence and burnout among ORNs in South Korea.	146 ORNs; gender: 86% female; age: 56% >30 years; education level: 61% bachelor’s degree; shift schedule: 56% 3 shifts; work experience: 47.3% >5 years Tertiary medical center, general hospitals; surgical area: NR	Interprofessional, personal	Communication competence, and resilience were linked to lower stress and burnout levels among ORNs. Females experienced higher job stress than males.
Li et al. (2021)*, China	Cross-sectional	To determine job stress among Chinese ORNs, test the mediating effect of burnout, and verify the moderating effect of over-commitment between job stress and mental health.	509 ORNs; gender: 473 female; age: *M* 35.4 years (*SD* 6.9); education level: 76% bachelor’s degree; shift schedule: NR; work experience: *M* 14.4 years (*SD* 7.7, range 1–34) Tertiary hospital; surgical area: NR	Personal	A majority of ORNs (70.3%) experienced job stress, thus negatively affecting organizational commitment through emotional exhaustion and depersonalization. Overcommitment moderated these effects, with job stress increasing burnout and reducing commitment. Emotional exhaustion also lowered commitment via depersonalization.
Li et al. (2022)*, China	Cross-sectional	To test the hypothesis that social support can moderate job strain on burnout and organizational commitment among ORNs (based on the job demand–control support model).	509 ORNs; gender: 92% female; age: *M* 35.4 years (*SD* 6.9, range 22–55); education level: 76% bachelor’s degree; shift schedule: NR; work experience: *M* 14.4 years (*SD* 7.7, range 1–34) Tertiary hospital; surgical area: NR	Interprofessional, personal	Good team skills were perceived to reduce or prevent stress. ORNs in the low social support group were more likely to experience depersonalization under job strain than those in the high social support group. Emotional exhaustion positively predicted depersonalization among all nurses.
Michael and Jenkins (2001), Australia	Cross-sectional	To describe the range and experiences of traumatic events reported by perioperative nurses.	233 ORNs; gender: 225 female; age: *M* 38 male, 41 female; education level: 51% hospital-based certificate; shift schedule: 54% day; work experience: *M* 11 years (range 1–37 years) Metropolitan hospital; surgical area: NR	Interprofessional	Exposure to traumatic events was reported by 69% of respondents and affected their well-being. The most reported traumatic event was verbal abuse from doctors.
Mitchell et al. (2011), UK	Semi-structured interviews	To identify critical nontechnical skills essential for safe and effective performance as an operating scrub nurse.	25 ORNs; gender: NR; age: NR; education level: NR; shift schedule: NR; work experience: *M* 15 years (*SD* 9.4) Type of hospital: NR; surgical area: NR	Interprofessional	SNs tried to remain calm and not panic others in the team. They recognized that it is sometimes difficult to prevent a stressful situation from being reflected in the manner of speech used toward other team members.
Naviaux et al. (2022), Belgium	Cross-sectional	To assess the stress factors affecting ORNs during the perioperative period.	612 ORNs; gender: 89.2% female; age: *M* 40.4 years (*SD* 9.9); education level: NR; shift schedule: NR; work experience: NR Private, public, and university hospitals; surgical areas: general, digestive, endocrine, orthopedic, transplantation, pediatric, neuro, and ENT	Interprofessional, work-related	Pressure to work quickly or without sufficient resources increased stress. The highest stress levels were due to the surgical team, particularly when a member was seen as incompetent or lacked confidence. Past relational issues with surgeons also increased stress. ORNs reported disruptive behaviors, with the most stressful being derogatory remarks, physical violence, threats, shouting, and degrading comments.
Olsen (1977), USA	Descriptive survey	To determine what ORNs perceive as stressful elements in their environment.	104 ORNs; gender: NR; age: NR; education level: NR; shift schedule: NR; work experience: NR State-funded and privately funded hospitals administered by a religious order; surgical area: NR	Interprofessional, work-related	As the length of experience increased, the stress decreased, while the stress from doctor’s abuse increased in everyone. Equipment did not work and disappeared, thereby contributing to perceived stress among ORNs.
Park et al. (2015), South Korea	Cross-sectional	To identify the prevalence and perpetrators of workplace violence against nurses and examine the relationship among work demands, trust, and justice in the workplace and the occurrence of violence.	139 ORNs; gender: female; age: *M* 28.6 years; education level: 76.6% baccalaureate or higher academic degree; shift schedule: NR; work experience: *M* 5.4 years University hospital; surgical area: NR	Interprofessional	In the OR, the physicians were the most frequent perpetrators of all types of violence, except for bullying. Violence by patients or patients’ families was rare among ORNs. ORNs were more likely to be exposed to violence from physicians.
Santamaria and O’Sullivan (1998), Australia	Cross-sectional	To identify the range of stressors encountered by perioperative nurses and investigate the relationship between the reported interpersonal stressors and the personality construct of lifestyle.	47 ORNs; gender: 41 female; age: *M* 31 years; education level: NR; shift schedule: NR; work experience: *M* 7.3 years Type of hospital: NR; surgical area: NR	Interprofessional	The main stressors for nurses were interpersonal interactions, organizational factors, and equipment. Interpersonal conflicts were the leading stressor, and the construct of lifestyle was significantly correlated with nurses’ psychological stress in specific interpersonal conflict situations.
Skråmm et al. (2021), Norway	Individual semi-structured interviews	To explore how ORNs experience OR team communication regarding nontechnical skills.	11 ORNs; gender: NR; age: *M* 52 years; education level: NR; shift schedule: NR; work experience: >5 years (as supervisors of ORN students) University hospital; surgical area: NR	Interprofessional	Surgeons being unprepared or demanding, and instruments different from those indicated in preoperative information, caused stress and frustration. Noise and brusque or poor communication also contributed to stress.
Sonoda et al. (2018), Japan	Cross-sectional	To evaluate ORNs’ sense of teamwork performance and level of mental stress to identify related factors.	375 ORNs (183 SN, 192 CN); gender: 96% female; age: *M* 31.2 years (*SD* 8.1, range 21–45); education level: NR; shift schedule: NR; work experience: 52% ≥4 years Type of hospital: NR; surgical areas: general surgery, orthopedic, gynecology, otolaryngology, neuro, urology, plastic, dermatology	Interprofessional, personal	Doctors were the key persons associated with scrub nurses experiencing mental stress. A sense of teamwork performance was associated with circulating nurses feeling mental stress. Moreover, 30%–40% of ORNs felt mental stress during surgery.
Tamer et al. (2022), Turkey	Cross-sectional	To determine the patient safety attitude perception of ORNs and nurse anesthetists and the factors affecting their perception levels.	60 ORNs; gender: 42 female; age: NR; education level: NR; shift schedule: NR; work experience: NR University hospital; surgical area: NR	Personal	A statistically significant difference was found between ORNs and nurse anesthetists’ groups in terms of age (*p* = .010), total experience (*p* = .006), job satisfaction (*p* = .015), and total score (*p* = .040). The perceptions of stress recognition were significantly higher in women than in men.
Teymoori et al. (2022), Iran	Individual semi-structured interviews, conventional content analysis	To identifying factors associated with occupational burnout from the perspective of Iranian ORNs.	18 ORNs; gender: 10 female; age: *M* 34 years (*SD* 5.9); education level: bachelor’s degree; shift schedule: variable shifts; work experience: *M* 11.16 years Governmental and nongovernmental hospitals; surgical area: NR	Interprofessional, work-related, personal	ORNs mentioned inadequate organizational support, inappropriate behavior from surgeons and colleagues, job-related stressors, and personal factors as contributors to burnout.
Tyler and Ellison (1994), England	Cross-sectional	To examine occupational stress in four areas of high-dependency nursing: theaters, liver unit, hematology/oncology and elective surgery.	60 ORNs; gender: 53 female; age: *M* 29.6 years; education level: NR; shift schedule: NR; work experience: *M* 9.78 years National Health Service hospital; surgical area: NR.	Work-related, personal	Stress from dealing with death and dying was much lower in OR than in any other department. Managing workload was most stressful for nurses in ORs.
Velando-Soriano et al. (2022), Spain	Cross-sectional	To identify risk and/or protection factors related to personality and depression variables involved in changes in the level of severity for each dimension of burnout syndrome for nurses in the surgical area and to quantify the effect of these factors on prognosis at various levels of each dimension.	214 ORNs; gender: 68.2% female; age: *M* 43.98 years (*SD* 9.01); education level: NR; shift schedule: 59.3% rotating shift; work experience: *M* 246.25 months (*SD* 116.19) Public health hospitals; surgical area: NR	Personal	Friendliness, responsibility, and extraversion protected against low personal accomplishment, while neuroticism was a risk factor. Friendliness protected against depersonalization, while depression increased its risk. Neuroticism, responsibility, and depression contributed to emotional exhaustion. The specific demands of surgical services and working conditions added stress, thereby impacting nurses’ adaptation and performance.
Vowels et al. (2012), USA	Descriptive survey	To examine the perceived amount of stress elicited by events in the perioperative environment, the frequency of those events, and their impact on the perceived stress of ORNs and OR technologists.	24 ORNs; gender: NR; age: NR; education level: NR; shift schedule: NR; work experience: NR Type of hospital: NR; surgical area: NR	Interprofessional, work-related	The top items ranked as having the highest stress impact scores among the ORNs were pressure to work more quickly, feeling unprepared for procedures, and inadequate communication among staff.
Wei et al. (2023), China	Cross-sectional	To investigate the professional mental stressors of ORNs to understand their job stress and mental health status.	171 ORNs; gender: 76% female; age: *M* 34.18 years (*SD* 10.22); education level: NR; shift schedule: NR; work experience: *M* 8.94 years (*SD* 5.06) Tertiary university hospital; surgical area: NR	Personal	Job pressure among ORNs was moderate. Significant differences were found in stress levels based on an ORN’s age, work experience, and professional rank and title. Gender did not significantly affect stress levels. Factors were positively correlated with the SCL-90 score, including the nursing profession and work, workload and distribution, working environment and resources, patient care and management, and interpersonal relationships.
Zhou et al. (2015), China	Cross-sectional	To explore the relationship between occupational stress and coping strategies among operating theater nurses in China and the factors influencing occupational stress and coping strategies.	65 ORNs; gender: 59 female; age: *M* 29.5 years (*SD* 7.4); education level: 90.8% advanced diplomas or below, shift schedule: 69.2% 2–4 nightshifts per month; work experience: 53.8% ≤9 years Teaching hospital; surgical area: NR	Interprofessional, work-related, personal	The most reported stressors included a heavy workload, making errors, low social status, and limited promotion opportunities. Stress was primarily related to workload/time pressure, professional/career challenges, patient care/interactions, interpersonal relationships and management issues, and resource and environmental problems. Higher stress levels were linked to fewer daily operations and day shifts. Night shift staff reported lower levels of stress. Older nurses without bachelor’s degrees experienced more stress related to resources and environment.

*Note.* ORN = operating room nurse; NR = not reported; OR = operating room; *M* = mean; *SD* = standard deviation; SN = scrub nurse; CN = circulation nurse; RN = register nurse; ENT = ear nose and throat.

* Two papers representing one study.

Overall, 3,957 ORNs were included in the papers. One paper did not report the number of participants (Hull et al., 2011), while two used the same sample (Li et al., 2021, 2022), which was counted once. Most participants were female, in a proportion ranging from 68% to 100% across the papers. The participants’ age ranged from at least 20 years to over 60 years. Experience in the field ranged from 2 months to over 30 years. Educational backgrounds were diverse, with most ORNs holding a diploma or bachelor’s degree in nursing. Certain papers described work schedule arrangements and reported a prevalent trend of rotating shifts. In addition, the examined papers described 17 surgical fields in which ORNs were employed. The ORNs involved in the studies were mainly employed in university or teaching hospitals.

### Thematic Groups

Data were organized into three thematic groups: (a) interprofessional factors contributing to occupational stress, (b) work-related factors influencing occupational stress, and (c) stress levels and personal factors influencing occupational stress among ORNs.

#### Interprofessional Factors Contributing to Occupational Stress

Team collaboration was a major source of occupational stress among ORNs (Aholaakko, 2011; Gillespie et al., 2013; Higgins & Macintosh, 2010; Holmes et al., 2020; Mitchell et al., 2011; Naviaux et al., 2022; Sonoda et al., 2018; Teymoori et al., 2022). Poor team skills, such as misunderstandings, incompetence, and lack of trust, contributed to such stress (Holmes et al., 2020; Naviaux et al., 2022). In addition, occupational stress among ORNs was reported to be influenced by several factors, including procedural challenges (e.g., bleeding), exposure to physical violence and emotional abuse, lack of peer support, and issues related to OR team composition (Almodibeg & Smith, 2021; Elfering et al., 2017; Gillespie et al., 2013; Higgins & Macintosh, 2010).

Occupational stress caused by relationships between surgeons and ORNs was often related to power relations and negative personal interactions (Aholaakko, 2011). ORNs frequently reported that collaboration with surgeons was a significant challenge (Ackah & Kwashie, 2023; Higgins & Macintosh, 2010; Michael & Jenkins, 2001; Naviaux et al., 2022; Olsen, 1977; Park et al., 2015; Santamaria & O’Sullivan, 1998; Sonoda et al., 2018; Teymoori et al., 2022). This was largely attributed to surgeons’ perceived aggressive and authoritarian behavior, which included shouting, making rude remarks and insults, displaying anger, and ignoring ORNs’ questions (Cook et al., 2001; Higgins & Macintosh, 2010; Teymoori et al., 2022). These situations were found to be further exacerbated by surgeons’ poor stress management skills and ORNs’ previous relational problems with surgeons (Naviaux et al., 2022; Teymoori et al., 2022).

Interactions among ORNs was also a source of their occupational stress (Michael & Jenkins, 2001; Teymoori et al., 2022). Unprofessional behavior (such as a lack of empathy and friendship) and negativity (such as slander, jealousy, and verbal abuse) were found to contribute to occupational stress for ORNs (Michael & Jenkins, 2001; Teymoori et al., 2022). In contrast, one paper found that ORNs perceived working conditions to be positive, characterized by solidarity and collaboration (Laflamme et al., 2019). Furthermore, ORNs who experienced low social support were more susceptible to depersonalization due to occupational stress than those with substantial social support (Li et al., 2022).

Communication styles were found to significantly influence the occupational stress among ORNs (Ackah & Kwashie, 2023; Cook et al., 2001; Higgins & Macintosh, 2010; Naviaux et al., 2022; Santamaria & O’Sullivan, 1998; Skråmm et al., 2021; Vowels et al., 2012). According to ORNs, certain surgeons established communication norms within the OR (Skråmm et al., 2021); as perceived by the ORNs, these norms often involved limited dialog, deficient communication skills, inadequate communication, verbal abuse, and delayed information sharing (Cook et al., 2001; Higgins & Macintosh, 2010; Naviaux et al., 2022; Skråmm et al., 2021; Vowels et al., 2012). Furthermore, factors such as environmental noise, language barriers, and a lack of timely communication regarding emergency cases exacerbated occupational stress (Ackah & Kwashie, 2023; Naviaux et al., 2022; Skråmm et al., 2021). Thus, enhancing communication competence played a potentially important role in protecting ORNs from burnout (E. Y. Lee et al., 2022).

#### Work-Related Factors Influencing Occupational Stress

A high workload, time pressure, and limited job control influenced occupational stress among ORNs (Ackah & Kwashie, 2023; Aholaakko, 2011; Akansel et al., 2019; Almodibeg & Smith, 2021; Bianchi, 2008; Eskola et al., 2016; Johnstone, 2000; Kawano, 2008; Naviaux et al., 2022; Olsen, 1977; Teymoori et al., 2022; Tyler & Ellison, 1994; Vowels et al., 2012; Zhou & Gong, 2015).

ORNs reported that there was an excessive daily volume of scheduled surgical procedures and that the time pressure within the OR contributed to their occupational stress (Ackah & Kwashie, 2023; Aholaakko, 2011; Bianchi, 2008; Johnstone, 2000; Naviaux et al., 2022; Vowels et al., 2012; Zhou & Gong, 2015). This time-related stress often stemmed from the fast-paced work environment coupled with high demands from surgeons, leaders, or ORNs themselves (Ackah & Kwashie, 2023; Aholaakko, 2011; Johnstone, 2000). This led to feelings of inadequacy in terms of meeting job requirements and ensuring patient safety (Aholaakko, 2011; Johnstone, 2000). Such stress was further intensified by factors such as staff shortages, the introduction of new technology, inadequate or faulty equipment, and a lack of necessary supplies (Ackah & Kwashie, 2023; Almodibeg & Smith, 2021; Johnstone, 2000; Naviaux et al., 2022; Olsen, 1977). Furthermore, circulating nurses faced the highest stress levels preoperatively because of the flurry of preparations. However, circulating nurses experienced reduced intraoperative stress, while scrub nurses reported lower stress levels both before and after surgeries compared to circulating nurses (Hull et al., 2011).

The work schedule and extended working hours contributed to the already high workload (Teymoori et al., 2022). Working in closed environments, particularly pressure during night shifts, exacerbated ORNs’ workload (Teymoori et al., 2022). However, one paper found that working with fewer surgical procedures per day and adhering to dayshifts correlated with higher stress levels (Zhou & Gong, 2015). Night shifts were associated with lower stress levels and improved work–life balance (Almodibeg & Smith, 2021; Kawano, 2008; Naviaux et al., 2022; Vowels et al., 2012).

In addition, low job control among ORNs also influenced their occupational stress levels (Naviaux et al., 2022; Teymoori et al., 2022). This lack of job control was linked to feelings of being unprepared, encountering unfamiliar procedures, and dealing with unpredictable events, all of which contributed to occupational stress among ORNs (Naviaux et al., 2022; Teymoori et al., 2022). Compared to nurses in other departments, ORNs exhibited lower job control scores (Kawano, 2008).

Other work-related factors that contributed to ORNs’ occupational stress were lack of coordination among departments, type of hospital, function of the working unit, work rewards, lack of administrative management, and administrative feedback (Akansel et al., 2019; Chen et al., 2009; Eskola et al., 2016; Teymoori et al., 2022).

#### Stress Levels and Personal Factors Influencing Occupational Stress Among ORNs

Although some studies suggested that ORNs may experience relatively lower levels of occupational stress compared to other healthcare professionals, they nonetheless reported considerable stress related to their work environment (Akgül & Aksoy, 2021; Cronin-Stubbs & Rooks, 1985; Kawano, 2008; Tamer et al., 2022). ORNs experienced moderate to high stress levels and reported symptoms such as emotional exhaustion, and depersonalization (Akansel et al., 2019; Almodibeg & Smith, 2021; Bianchi, 2008; Chen et al., 2009; Elfering et al., 2017; Eskola et al., 2016; Li et al., 2022; Sonoda et al., 2018; Wei et al., 2023). Such stress affected ORNs’ organizational commitment, primarily through the mechanisms of emotional exhaustion and depersonalization, thus leading to reduced organizational commitment and burnout (Li et al., 2021). Facing stress, ORNs with low over-commitment were more likely to have emotional exhaustion, while those with high over-commitment were more likely to have low organizational commitment (Li et al., 2021). Psychosocial factors of the OR environment, including the nursing profession, workload, resources, patient care, management, and interpersonal relationships, were associated with high levels of adverse mental health symptoms among ORNs (Wei et al., 2023). In contrast, two papers found that ORNs positively perceived their work environment, thus contributing to their overall job satisfaction (Eskola et al., 2016; Tamer et al., 2022).

Patient safety and moral distress were sources of occupational stress among ORNs influenced by ethical dilemmas and emotional involvement with patients (Ackah & Kwashie, 2023; Aholaakko, 2011; Bianchi, 2008; Chen et al., 2009; Teymoori et al., 2022; Zhou & Gong, 2015). ORNs experienced situations such as poor patient prognoses, death, trauma, and the implications of patient care decisions (Ackah & Kwashie, 2023; Aholaakko, 2011; Bianchi, 2008; Chen et al., 2009; Teymoori et al., 2022; Zhou & Gong, 2015). The emotional aftermath of traumatic events had a strong impact on ORNs, reducing their ability to function properly for days after such events (Teymoori et al., 2022). ORNs felt a deep sense of responsibility for patients, which occasionally led to moral stress, as their focus and priorities did not always align with those of surgeons (Aholaakko, 2011). However, in contrast, one study found that stress from dealing with death and dying was much lower in the OR than in other departments (Tyler & Ellison, 1994).

Adapting to a stressful, power-related, and demanding OR environment requires a certain personality type, characterized by strength of character (Laflamme et al., 2019; E. Y. Lee et al., 2022; Velando-Soriano et al., 2022). ORNs emphasized the importance of being able to stand up for oneself, endurance, and showing initiative (Laflamme et al., 2019). Personality traits such as friendliness, responsibility, and extraversion functioned as protective factors in the OR, particularly in achieving personal accomplishments and preventing depersonalization (Velando-Soriano et al., 2022). Certain behaviors, such as difficulty talking about issues and worrying, contributed to stress among ORNs (Ackah & Kwashie, 2023). Furthermore, increased resilience among ORNs was associated with lower levels of burnout (E. Y. Lee et al., 2022), while traits such as neuroticism, responsibility, and depression were considered risk factors that could lead to emotional exhaustion (Velando-Soriano et al., 2022).

A variety of demographic variables, such as age, work experience, educational level, gender, health status, family, and financial circumstance, influenced occupational stress levels among ORNs (Ackah & Kwashie, 2023; Akansel et al., 2019; Eskola et al., 2016; E. Y. Lee et al., 2022; Olsen, 1977; Tamer et al., 2022; Wei et al., 2023; Zhou & Gong, 2015). ORNs with 5 to 9 years of experience reported higher stress levels than those with shorter or longer tenure (Eskola et al., 2016). In contrast, longer experience was also related to lower stress (Olsen, 1977) or found no correlation (Zhou & Gong, 2015). Further, ORNs with bachelor’s or diploma degrees experienced more stress than those with lower education (E. Y. Lee et al., 2022; Olsen, 1977), although bachelor’s degree holders revealed lower burnout and emotional exhaustion (E. Y. Lee et al., 2022). Gender-based findings were mixed. Two studies reported higher stress among women (E. Y. Lee et al., 2022; Tamer et al., 2022), while another found no difference (Wei et al., 2023). Additionally, chronic illness, family duties, and financial issues also contributed to stress among ORNs (Ackah & Kwashie, 2023; Akansel et al., 2019; E. Y. Lee et al., 2022). However, one study found that ORNs’ demographic variables were not related to their total stress scores (Akansel et al., 2019).

## Discussion

Our findings reveal that high workloads and collaboration between ORNs and surgeons are major sources of occupational stress. High workload is influenced by the volume of surgical procedures, time constraints, and challenges related to staffing, technology, and equipment. The OR environment also contributes to moral distress, primarily related to patient care and ensuring patient safety. Despite these challenges, there is a lack of research on how the specific personality traits of ORNs influence these stressors and the overall dynamics of the OR.

Our findings suggest that ORNs often experience abusive behavior from surgeons, which includes derogatory remarks, physical threats, and degrading comments, thereby contributing significantly to occupational stress in ORNs. Moreover, previous reviews have highlighted the hierarchical structures in the OR environment in which surgeons tend to dominate and exhibit disruptive behavior more frequently than other OR personnel, while ORNs show deference (Chrouser et al., 2018; Jacob, 2015; H. Lee et al., 2023). ORNs appear to be a target for surgeons’ release of frustrations during surgery and receive little recognition for their role, frequently being reprimanded in front of others and treated merely as assistants (Jacob, 2015; James-Scotter et al., 2019).

However, the ORN–surgeon relationship is particularly important for ORNs’ job satisfaction (Jacob, 2015; James-Scotter et al., 2019). Recognition by surgeons for job performance is associated with improved performance and increased confidence in speaking up and feeling more valued by other OR personnel (James-Scotter et al., 2019). Good ORN–surgeon collaboration involves equal knowledge-sharing and mutual respect for each other’s opinions (Jacob, 2015). Furthermore, the OR environment frequently lacks the psychological safety required for effective team collaboration (Chrouser et al., 2018). Due to work pressure, unique culture, and isolation of the OR, conflicts and disruptive behavior occur more frequently; such behavior is often more tolerated in an OR than in other parts of the hospital (Chrouser et al., 2018). According to Karasek and Theorell’s (1990) job demand–control–support model, social support moderates the negative effects of high job demands and low control, acting as a buffer that may reduce stress.

Our review indicates that the high workload among ORNs contributes to occupational stress. The OR is a highly regulated environment that is meticulously organized to achieve the maximum number of surgical procedures by controlling space and time (Jacob, 2015). The timeframe for surgery schedules includes preparation of the patient and instruments, operating time, and patient recovery before transfer to the recovery room (Jacob, 2015). ORNs are involved in patient care from the moment the patient arrives in the surgery department until they are transferred to the recovery room and the completed treatment has been evaluated (Blomberg et al., 2015). In contrast, surgeons have a specified amount of time in the OR and may perceive the surgical timeframe as the period from the incision until the final suture is placed (Bull & Fitzgerald, 2004). This discrepancy could lead to a conflicting understanding of the actual surgical time and the “surgeons’ time.” ORNs strive to reconcile different perceptions of this timeframe, caught between the surgeon’s demands and job responsibilities (Bull & Fitzgerald, 2004). Furthermore, ORNs face great pressure to complete their work quickly and devote most of their time to surgical procedures. However, their workload can fluctuate due to patient conditions, time pressures from tight schedules, or interruptions (Chrouser et al., 2018).

This review reveals that leadership plays an important role in the OR environment, as ORNs highlighted several contributors to heavy workload stress, including inadequate staffing, insufficient material resources, technological challenges, and functioning of the unit. While more patients are now receiving care due to technological and procedural improvements, the fragmented nature of health systems often hampers the communication and collaboration necessary for the provision of high-quality care (Figueroa et al., 2019). This fragmentation is exacerbated by increasing specialization and disassociation among health personnel and leads to poor human and technical resource allocation, thereby creating a mismatch between demand and supply (Figueroa et al., 2019). This may affect the quality of patient care and lead to high ORN turnover, thus making it challenging to maintain a supportive environment for ORNs. Moreover, supportive leadership behavior positively influences nurses’ sense of personal accomplishment and burnout levels (Mudallal et al., 2017). Support from and recognition by leaders is important for job satisfaction (James-Scotter et al., 2019). Hence, leaders who involve nurses in decision-making processes, grant them autonomy, make work more meaningful, and express confidence in their performance can reduce emotional exhaustion and depersonalization among ORNs (Mudallal et al., 2017).

Further, our findings indicate considerable work-related moral stress among ORNs due to knowledge of a patient’s poor prognosis, patients dying during surgery, and handling patients with acute conditions, such as trauma. The emotional impact of witnessing such situations and the challenge of managing them exacerbate ORNs’ feelings of stress and lead to burnout. Similarly, Salari et al. (2022) found that nurses experience high levels of moral stress due to their daily encounters with dilemmas and emotionally demanding situations. An important aspect of ORNs’ work is engaging in respectful care and being patients’ advocates during surgery (Sirevåg et al., 2023). This is reflected in their dedication to upholding the plans and standards for various types of patients and potential situations that may occur during a surgical procedure (Chellam Singh & Arulappan, 2023; Sirevåg et al., 2023). However, moral stress arises when nurses know the ethically appropriate course of action but face barriers, such as organizational pressures, regulatory constraints, or interpersonal conflicts, that prevent them from acting accordingly (Salari et al., 2022). According to Lazarus and Folkman (1984), stress appears when there is a perceived discrepancy between the demands of the situation and the individual’s resources to cope with these demands. Thus, ORNs who experience moral distress need support and guidance to manage their feelings and maintain their professional integrity and well-being (Salari et al., 2022).

According to the person–environment fit model, the relationship between personal traits and the work environment affects stress and strain among ORNs (French et al., 1982). However, this review identified limited research on how specific personality traits influence ORNs’ ability to adapt to the OR environment. Consequently, future research should explore the role of individual personality traits in how ORNs cope with occupational stress. Investigating these factors may provide deeper insight into personalized approaches to occupational stress reduction.

Our review did not identify any interventions designed to reduce occupational stress among ORNs. Most studies utilized cross-sectional designs (*n* = 22), thus offering only a snapshot of occupational stress in ORNs at a single time point. This limits the understanding of how psychosocial factors and stress levels evolve and change over time. Future studies should adopt a longitudinal design to track changes in psychosocial factors and stress levels, thereby providing insights into long-term outcomes and trends. Future research should also develop and evaluate the effectiveness of interventions to reduce occupational stress in the OR. Employing mixed methods could provide a more comprehensive understanding of the factors that influence occupational stress among ORNs.

This review revealed that 39 different measurement instruments were used in the papers, thus illustrating large heterogeneity in the assessment of occupational stress. Thus, a more consistent and standardized approach to measuring psychosocial factors that influence occupational stress must be adopted. Future studies should utilize the same instrument to ensure comparisons and consistent data across studies and countries, thereby facilitating a more comprehensive understanding of how these factors influence stress among ORNs in different cultural and health care system contexts.

Our review found that eight studies examined the influence of demographic variables, experience, educational level, gender, and health status on occupational stress, but these had conflicting findings. Therefore, future studies should examine the interplay between demographic and psychological factors that cause occupational stress, including exploring why certain groups report different stress levels.

Moreover, most participants were women, which reflects the global gender distribution within the nursing profession. Consequently, our review findings may be representative of female ORNs and, notably, suggest that female ORNs report higher levels of stress than male ORNs. However, this review may have overlooked the unique stressors faced by male ORNs, which should be explored in future research.

This review has several strengths. The review protocol was published and adhered to a recognized framework, thereby ensuring methodological rigor and transparency. The search strategy was developed with assistance from an experienced librarian to enhance the accuracy and comprehensiveness of systematic database searches. Study selection and data extraction were independently conducted by pairs of researchers, thus ensuring reliability. To further enhance the validity and reliability of the analysis, researcher triangulation was employed, leveraging different competencies and backgrounds to promote alternative interpretations in the analysis and interpretation of the findings.

This review also has several limitations. We may not have been able to identify all the relevant search terms for ORNs, and only studies in English and Scandinavian were included. Consequently, it is possible that all relevant papers were not included. Moreover, the geographic diversity may make it challenging to generalize the findings due to the vast differences in psychosocial factors across countries due to variations in cultural norms and health care policies. In addition, a small deviation from the registered protocol (https://osf.io/9vdqt) occurred as studies conducted exclusively during the COVID-19 period were excluded, as the extraordinary working conditions were not considered representative of the typical working environment for ORNs. Finally, the variation in measurement instruments used to assess occupational stress and psychosocial factors complicates not only the comparison of findings across different studies but also the analysis and interpretation of results due to a lack of standardization.

This scoping review systematically mapped psychosocial factors that influence occupational stress among ORNs. These factors predominantly include ORNs’ interpersonal challenges while working with surgeons and the associated high workload. Work-related moral distress was influenced by the intricate dynamics of patient care and the imperative of ensuring patient safety. Notably, there is a research gap, particularly regarding how ORNs’ individual personality traits interact with and influence occupational stress.

### Implications for Occupational Health Nursing Practice

Understanding the psychosocial factors influencing occupational stress among ORNs is important for ORN educators and ORN leaders to improve working conditions in OR. To address the identified challenges of high workload and interprofessional tensions, clinical practice should prioritize the development and implementation of targeted interventions. These could include leadership training focused on supportive and relationship-oriented management as well as team-building initiatives aimed at fostering mutual respect and understanding between ORNs and surgeons. Stress management programs may also be implemented to enhance ORNs’ coping strategies and resilience. Additionally, organizational measures, such as improved scheduling and adequate staffing, may reduce time pressure and the perceived imbalance between demands and available resources.

**In Summary**

• Recognizing that operating room nurses face high demands and complex responsibilities, this scoping review aimed to map existing research on the psychosocial factors influencing occupational stress in these nurses’ working environments.
• The review identified recurring stressors, such as interprofessional tensions, moral distress, and time pressure. These findings emphasize how complex workplace interactions and organizational demands impact operating room nurses’ mental health and job performance.
• This review highlighted a lack of research on how the personality traits of operating room nurses could influence their stress perception.
• This scoping review contributes to occupational health and environmental nursing by identifying areas for targeted interventions such as improved leadership support, enhanced team collaboration, and increased attention to individual coping needs.

## Supplemental Material

sj-docx-1-whs-10.1177_21650799251377451 – Supplemental material for Psychosocial Factors That Influence Occupational Stress in Operating Room Nurses’ Working Environment: A Scoping Review Supplemental material, sj-docx-1-whs-10.1177_21650799251377451 for Psychosocial Factors That Influence Occupational Stress in Operating Room Nurses’ Working Environment: A Scoping Review by Vibeke Tjugum, Lena Rengård Kolstad, Marie Hamilton Larsen and Simen A. Steindal in Workplace Health & Safety
