# Supplementary material for: Psychosocial Factors That Influence Occupational Stress in Operating Room Nurses’ Working Environment: A Scoping Review
Source: Workplace Health Saf. 2025 Nov 6;74(3):107–23. doi: 10.1177/21650799251377451 (PMC12861551; doi:10.1177/21650799251377451)
Supplement: sj-docx-1-whs-10.1177_21650799251377451 – Supplemental material for Psychosocial Factors That Influence Occupational Stress in Operating Room Nurses’ Working Environment: A Scoping Review [file sj-docx-1-whs-10.1177_21650799251377451.docx]

**Supplementary File 1.** Deviations from the Study Protocol

| **Planned approach described in the protocol** | **Type, description and justification of the**  **deviation from the protocol** |
| --- | --- |
|  |  |
| Psychosocial factors that influence occupational stress or similar concepts, such as work-related stress, job stress and stress outcomes.  Psychological factors are related to how ORNs handle experiences of their work situation, work content and work tasks, both cognitively and emotionally.  Social factors are ORNs’ interpersonal interactions at work. | Studies that solely focused on the daily work of ORNs during the Covid-19 pandemic were excluded. This was due to the significant alterations in ORNs’ work tasks during the pandemic resulting from factors such as increased workload, limited resources and equipment and an upsurge in patient numbers. These exceptional circumstances do not accurately reflect the typical working environment for ONRs. |
|  |  |
| We planned to use Microsoft Word for data extraction from the included papers. | We used Microsoft Excel for data extraction from the included papers. |
|  |  |
| We had initially not planned to use any software tool for data analysis | We used NVivo to identify patterns and  systematically organise data into thematic groups. |
|  |  |
| In the initial stages of the study, librarian Eline Kaupang Petersen was listed as one of the co-authors of the manuscript | During the course of the project, a change in her workplace limited her further involvement in the study. It was determined that her contributions were more appropriately recognised in the acknowledgements section. |
